# Death of Dr. Zagorsky

**Published:** 1849-05

**Authors:** 


We find in the Gazette des Hopitaux the following items of medical intelligence.

Doctor Zagorsky, celebrated by his works upon anatomy and physiology, died a short time since at St. Petersburg. He was a member of the Academy and professor in the Medico-Chirurgical Society of St. Petersburg. Born on the 9th of August 1756, at Tscheraigon, he was appointed prosector in Anatomy in 1787, he was called to the chair of Anatomy and Physiology in the School of Medicine at Moscow, which he afterwards left to fill the same post at St. Petersburg.

